# Breast MRI texture analysis for prediction of BRCA-associated genetic risk

**DOI:** 10.1186/s12880-020-00483-2

**Published:** 2020-07-29

**Authors:** Georgia Vasileiou, Maria J. Costa, Christopher Long, Iris R. Wetzler, Juliane Hoyer, Cornelia Kraus, Bernt Popp, Julius Emons, Marius Wunderle, Evelyn Wenkel, Michael Uder, Matthias W. Beckmann, Sebastian M. Jud, Peter A. Fasching, Alexander Cavallaro, André Reis, Matthias Hammon

**Affiliations:** 1 Institute of Human Genetics, University Hospital Erlangen, Friedrich-Alexander-Universität Erlangen-Nürnberg, Schwabachanlage 10, 91054 Erlangen, Germany; 2 Siemens Healthcare, Imaging Analytics Germany, 91054 Erlangen, Germany; 3 Department of Radiology, University Hospital Erlangen, Friedrich-Alexander-Universität Erlangen-Nürnberg, 91054 Erlangen, Germany; 4 Department of Gynecology and Obstetrics, University Hospital Erlangen, Comprehensive Cancer Center Erlangen-EMN, Friedrich-Alexander-Universität Erlangen-Nürnberg (FAU), 91054 Erlangen, Germany

**Keywords:** Breast cancer, HBOC, MRI, Texture analysis, *BRCA1/2*, L-PCR

## Abstract

**Background:**

*BRCA1/2* deleterious variants account for most of the hereditary breast and ovarian cancer cases. Prediction models and guidelines for the assessment of genetic risk rely heavily on criteria with high variability such as family cancer history. Here we investigated the efficacy of MRI (magnetic resonance imaging) texture features as a predictor for *BRCA* mutation status.

**Methods:**

A total of 41 female breast cancer individuals at high genetic risk, sixteen with a *BRCA1/2* pathogenic variant and twenty five controls were included. From each MRI 4225 computer-extracted voxels were analyzed. Non-imaging features including clinical, family cancer history variables and triple negative receptor status (TNBC) were complementarily used. Lasso-principal component regression (L-PCR) analysis was implemented to compare the predictive performance, assessed as area under the curve (AUC), when imaging features were used, and lasso logistic regression or conventional logistic regression for the remaining analyses.

**Results:**

Lasso-selected imaging principal components showed the highest predictive value (AUC 0.86), surpassing family cancer history. Clinical variables comprising age at disease onset and bilateral breast cancer yielded a relatively poor AUC (~ 0.56). Combination of imaging with the non-imaging variables led to an improvement of predictive performance in all analyses, with TNBC along with the imaging components yielding the highest AUC (0.94). Replacing family history variables with imaging components yielded an improvement of classification performance of ~ 4%, suggesting that imaging compensates the predictive information arising from family cancer structure.

**Conclusions:**

The L-PCR model uncovered evidence for the utility of MRI texture features in distinguishing between *BRCA1/2* positive and negative high-risk breast cancer individuals, which may suggest value to diagnostic routine. Integration of computer-extracted texture analysis from MRI modalities in prediction models and inclusion criteria might play a role in reducing false positives or missed cases especially when established risk variables such as family history are missing.

## Background

Hereditary breast and ovarian cancer (HBOC) accounts for 5–10% of all breast cancer cases. Approximately 15–24% of familial cases are attributed to germline deleterious variants in the two tumor suppressor genes, namely *BRCA1* and *BRCA2* (hereafter referred as *BRCA1/2*) [1–3]. A recent large prospective study showed a lifetime-risk for breast cancer development by the age 80, to be 72% for carriers of a pathogenic variant in *BRCA1* and 69% for *BRCA2*, respectively [4]. The assessment of genetic cancer risk and subsequently the selection for genetic screening in Germany is based on guidelines and selection criteria that evaluate the empirical probability (EP) for the identification of *BRCA1/2* variants. This is calculated by taking into consideration primarily the family history of breast and ovarian cancer, the age at disease onset and the identification of bilateral/contralateral breast tumors, and should exceed 10% [2, 5]. A triple-negative breast tumor (TNBC; no expression of estrogen, progesterone and HER2 receptors) regardless of the family history and age at diagnosis is also considered as inclusion criterion due to the high probability of pathogenic variant detection (*BRCA1/2* variants in 11.2–18.3% of the cases) [6–8]. Furthermore, a number of prediction models have been developed to assess the likelihood of a *BRCA1/2* variant detection, mainly by taking into consideration the family cancer history of an affected individual [9–11]. Nevertheless, information about family structure is often limited and genetic screening inclusion criteria are subjected to the personal judgment of clinicians often leading to exclusion of many affected individuals with genetic predisposition from testing [12, 13].

Population-based gynecological screening as well as preoperative control of women with breast cancer is mainly based on ultrasound and mammography. Over the last years breast magnetic resonance imaging (MRI) is increasingly used as a supplemental imaging modality in newly diagnosed breast cancer individuals prior to therapy. It facilitates an accurate detection, preoperative staging and monitoring of the tumor resulting in a more efficient planning of personalized treatment strategy [14, 15].

Many reports comparing MRI morphologic and kinetic features between *BRCA1/2*-related and sporadic tumors have been published. *BRCA* tumors, especially the *BRCA1*-associated, more frequently show benign fibroadenoma- or cyst-like features as compared to the sporadic ones. These include well-defined, oval and round shaped masses with smooth, pushing margins and homogenous internal enhancement [16–22]. They also exhibit high T2 signal intensity, reminiscent of benign or cystic lesions, in contrast to the low or intermediate signal intensity usually found in sporadic breast cancers [19]. Moreover, benign MRI kinetic features such as slow or intermediate early rise and persistent enhancement in the delayed phase has been demonstrated in 33% of tested individuals with high genetic risk or *BRCA1/2* alterations [17]. Finally, the majority of *BRCA1/2* breast cancers showed a higher rim enhancement, an imaging feature associated with aggressive malignant tumors [20, 21, 23]. Other studies though, could not identify an association between MRI features and *BRCA* mutation status [24, 25]. A significant limitation of the aforementioned studies regardless of the results is that the interpretation of MR images was based on the manual review of radiologists, who were already aware of the molecular findings.

Over the last years an innovative medical imaging analysis, referred to as imaging texture analysis or radiomics, has been developed [26]. This enables not only the interpretation of macroscopic radiological features, but also the computer-based extraction of hidden-to-the-naked-eye textures and shape features from radiographic images. Texture analysis roughly includes the following steps: a) Acquisition of high quality radiological imaging b) identification and manual or automated segmentation of the lesion of interest, c) extraction of large amounts of quantitative imaging features and d) analysis using statistical models. Radiomics allowed the association of image traits with phenotypes, tissue characteristics, genomic signatures and protein expression patterns of a tumor [26–30]. To date, analysis of breast MRI textural features has been applied for the discrimination between malignant and benign lesions [31–35], correlation with tumor histological and molecular subtypes [36–40] and even prediction of chemotherapy response [41].

An association between MRI texture features alone or coupled with non-imaging variables and *BRCA1/2* genetic risk has not been previously examined. A strong relationship could highlight the information extracted from MRI as an additional selection variable for subsequent genetic screening. Here we present results from a pilot study aimed at quantifying the efficacy of the breast MRI phenotype as a potential predictor relevant to *BRCA*-related breast cancer.

## Methods

### Study cohort

Clinical and genetic data of female breast cancer individuals at high genetic risk referred for diagnostic purposes to our interdisciplinary outpatient clinic were retrospectively collected. All fulfilled the criteria of the German Consortium for Hereditary Breast and Ovarian Cancer for diagnostic genetic screening. Higher EP for identifying *BRCA1/2* pathogenic variants was calculated, when the family history including the index case consisted of: i) at least one breast and one ovarian cancer case (48.4%), ii) at least 3 breast cancer cases, with two of them manifesting before the age of 51 (30.7%) iii) bilateral/contralateral breast cancer by the index case with the first tumor diagnosed before the age of 51 (24.8%) iv) at least 3 breast cancer cases regardless of the age at diagnosis (22.4%) [2]. In case of pedigrees with one affected breast cancer relative and bilateral breast cancer in the affected individual we considered three independent breast tumors and calculated an EP of 30.7%. Triple negative tumors regardless of the family history were also included (EP 11.2%) [6].

The initial study cohort consisted of 186 affected individuals at high risk of HBOC (empirical probability > 10%). Informed written consent was obtained from all patients. The study was approved by the Ethical Committee of the Medical Faculty of the Friedrich-Alexander-Universität Erlangen-Nürnberg. Genetic screening at the time of diagnosis or during the aftercare identified pathogenic variants in either *BRCA* gene in 92 cases, whereas in the remaining 94 no alteration was detected (control group). Individuals with alterations assessed as variants of unknown significance (VUS) in *BRCA1/2* or other breast cancer susceptibility genes were excluded from the analysis. MRIs at the time of diagnosis and before the initiation of treatment were available for 94 of the aforementioned women. After excluding cases with an MRI acquisition protocol which did not fulfill the standards for the imaging texture analysis (e.g. no suitable machine and/or machine settings/protocols, insufficient magnetic field strength and resolution), the final number of eligible breast cancer individuals for this study was 41: 16 with a *BRCA1/2* deleterious variant (13 *BRCA1* and 3 *BRCA2*) and 25 without (controls) (Fig. S1). In total 134 MRIs from all the cases studied were available. Clinical data of the final study cohort including the age at disease onset and unilateral or bilateral/contralateral cancer, histopathological information about the receptor status and detailed family cancer history with 1st, 2nd and 3rd degree affected relatives as well as the calculated EP for identifying a *BRCA1/2* variant are summarized in supplementary Table S1.

### *BRCA1/2* screening

DNA from peripheral blood lymphocytes was extracted with an automated chemagic MSM I system according to standard procedures (Perkin Elmer, Baesweiler, Germany). Mutational analysis of *BRCA1/2* genes was performed either with Sanger sequencing and MLPA analysis for copy number variant (CNV) identification (MRC-Holland, Amsterdam, Netherlands) or with Next Generation Sequencing on a MiSeq platform (Illumina, San Diego, CA). The commercially available targeted resequencing kit, TruSight Cancer Sequencing Panel (Illumina, San Diego, CA), was used according to the manufacturers’ instructions. Sequencing reads were aligned and processed following standard clinical grade genetic diagnostics as previously described [42]. The targeted genes had an average coverage of 400 reads. Complete coverage (> 30 reads) was obtained for the coding regions and the 10 bp of flanking intronic regions.

### MRI acquisition

Breast magnetic resonance imaging was performed with a 1.5 T scanner in the prone position (Avanto, Siemens Healthcare, Erlangen, Germany), using a dedicated coil. A routine scan protocol was performed, including axial 3D fat-suppressed fast low angle shot T1-weighted sequences (fl3d). After one unenhanced sequence, 6 ml of gadolinium-based contrast medium (Gadovist, Bayer AG, Leverkusen, Germany) were injected and five post-contrast sequences were acquired (in-plane spatial resolution 0.75 × 0.75 mm, repetition time 7.58 ms, echo time 4.78 ms, slice thickness 1.5 mm, flip angle 20 deg, FOV 340 mm, matrix 448 × 331). For feature extraction the first subtracted 3D fat-supressed fast low angle shot transverse T1-weighted sequence (the unenhanced T1-weighted sequence was subtracted from the identical sequence performed after gadolinium administration).

### Segmentation and imaging feature extraction

The DICOM-files of MR images were displayed with a dedicated software (syngo plaza, Siemens Healthcare, Erlangen, Germany) and anonymized using the “Incognito” algorithm (last reviewed 2018-09-26) [43]. Next, they were converted into mhd-format images. Breast cancer lesions in the 3D images were manually segmented via the open-source program MITK (last reviewed 2018-02-28) [44]. The accuracy of each annotation was reviewed and corrected if necessary, by one or more clinical radiologists. The extraction of breast lesion image samples from the T1-weighted MR images comprised three main steps: first, the malignant lesions were manually annotated in the 3D images, and then stored as binary masks (Fig. S2). Secondly, a sampling of points in the image regions that contain these malignant lesions was performed. Finally, “image patches” were manually created around the sampled points of the previous step.

**Fig. 1:**
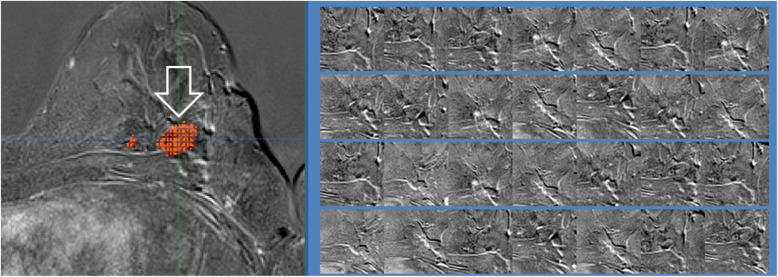
Illustration of lesion annotation mask and intensity patches Left, lesion annotation masks depicted in red and marked by a white arrow used to create intensity patches around the selected locations. Right, visualization of intensity patches

In detail, the lesion annotation masks from the first step were used to define regions of interest (ROI) in their associated MR images. Within those regions, random locations were chosen with a uniform distribution (Fig. S3A). Locations extracted from images of women having a *BRCA1/2* mutation were referred to as positive samples, while those stemming from controls were called negative samples. About 1000 samples were created from each image and a ratio of 1:1 for positives and negatives was enforced. The samples extracted were used as seed points, i.e. each sample defined the center point around which MR image intensity “patches” were extracted. These patches provided local views of the lesions to the training system. In our setup, the patch size was chosen to be 65 × 65 voxels. The image intensities were normalized to values between 0 and 1 in order to construct the patches (Fig. 1 and Fig. S3B).

### Non-imaging features

Several non-imaging features were also used to complement the extracted imaging components (see also supplementary Table S1). These include two clinical variables: age at disease onset (Age) and bilateral/contralateral breast cancer (BBC: 1 for positive, 0 for negative); seven family cancer history variables: number of 1st degree relatives with breast cancer (FDR.BC), number of 1st degree relatives with ovarian cancer (FDR.OC), number of 2nd degree relatives with breast cancer (SDR.BC), number of 2nd degree relatives with ovarian cancer (SDR.OC), number of 3rd degree relatives with breast cancer (TDR.BC), number of 3rd degree relatives with ovarian cancer (TDR.OC), male breast cancer (MBC) and triple negative breast cancer (TNBC: 1 for positive, 0 for negative).

### Lasso-principal component regression (L-PCR)

We set up four different classification problems to assess: (i) The relative predictive performance of either the TNBC histology, clinical variables and family history variables, or imaging features [T1* weighted values from 4225 voxels sized 0.75 × 0.75 × 3.7 mm from the annotated region of interest] regarding the *BRCA* genetic risk estimation, (ii) whether imaging components could add to the predictive value of the non-imaging variables and to which extend, (iii) whether imaging components could compensate missing data about cancer history of distant (2nd or 3rd degree) relatives, and finally (iv) whether imaging components have a similar predictive value as family cancer history. For those classification problems with two or less parameters we fit a standard logistic regression with no model penalization, while for those where the number of parameters was > 2, we fit a lasso-penalized logistic regression. All models using imaging data were prefaced by a principal component analysis (PCA) transformation and the resulting principal component scores were fed to the lasso-estimator. Specifically, we computed PCA on the full set of 4225 imaging variables extracted from the annotated ROIs such that 95% of the total variance in the image ROIs were explained. The surviving 41 principal components were either entered directly into the L-PCR estimator or augmented with subsets of non-imaging variables, depending on the classification problem in question. Since 41 parameters in 41 subjects would lead to a poorly determined and unreliable solution to the logistic-least squares problem, we penalized the regression solution using the lasso penalty. In general, the lasso penalty enforces a highly sparse solution upon the estimated regression, allowing us to identify a small subset of imaging principal components that should best discriminate between the two groups (Fig. S4). Our model-fitting procedure is described in more detail in the following section. All methods were implemented using the R framework (3.4.1) [45], the Caret package [46] and the glmnet toolbox [47]. The lasso penalty [47, 48] compensates for the large number of variables relative to the number of subjects, choosing only the most relevant components to the classification problem (See also supplementary Methods).

### Model fitting and hyper-parameter estimation

Lasso regression requires choosing a tuning or hyper-parameter (Lambda) [47, 48], which weights the influence of the penalization term on the model fidelity or goodness-of-fit term (see also Fig. S5 and supplementary Methods). Lambda is selected to optimize the out of sample model performance fit, while protecting against overfitting. In practice Lambda is selected through an iterative grid search in the outer loop of a cross-validation procedure [47, 48]. We searched for an optimal Lambda between 0 (standard regression) and 3 (empirically chosen) using a resolution of 0.05 and repeatedly fit and tested the model on each cross-validation fold for each choice of Lambda. Specifically, for each possible choice of Lambda, we assessed model performance in the following manner: First we split the data at random into training/testing subsets by using repeated *k*-fold cross-validation with *k* = 5, leading to a data subset that used 80% of the original data to train the regression model, and 20% of the data to be used to assess out-of-sample model prediction performance. In standard *k*-fold cross-validation, uncertainty in performance estimates may both be reduced and information about the precision of those estimates obtained by repeating. We repeated each *k*-fold cross-validation *L = 20* times and averaged across the *L* estimates returned from each single *k*-fold. Within each of the *k*-folds, one iteration proceeded as follows: we fit the model to the training subset, and estimated the out-of-sample AUC from the held-out 20% subset. Next the held-out data was returned to the main dataset and the process repeated until each subset of the data had been used in both model training and in assessing its out-of-sample performance on the unseen data subset. The average across the five AUC estimates was reported as the overall cross-validation estimate of performance for a particular repeat. At the end the AUC measure was averaged across the *L* repeats and placed on the location on the tuning grid corresponding to that choice of Lambda. The net result was a set of cross-validation error curves and their associated standard errors plotted as a function of tuning parameter. We selected the optimal point on these curves yielding maximal model performance and plotted the corresponding receiver operating characteristic (ROC) for the optimal lasso classifier for each of the data classification problems. Model predictive performance was assessed by using AUC. In all performed experiments AUC can be interpreted as an estimate of the probability of the classifier ranking a randomly chosen *BRCA1/2* carrier higher than a randomly chosen control.

## Results

### Prevalence of clinical data in the studied cohort and comparison with the literature

The mean age of disease onset did not differ significantly between both groups and ranged from 36.8+/− 7 years for the *BRCA* carriers and 38+/− 11 years for the controls (t-Test, *p*-value: 0.68). To examine whether the genetic and clinical data in this cohort exhibit any difference compared to the literature, two-sided binomial tests were performed. The contribution of *BRCA1* (13 of 16) and *BRCA2* (3 of 16) pathogenic variants in HBOC cases in the current study was not significantly different (binomial *p*-value: 0.292) with that reported in literature (*BRCA1*: 66% and *BRCA2*: 34%) [49, 50]. In our cohort, 12 of 13 cases with *BRCA1* pathogenic variants and 5 of 21 (23.8%) of the controls developed a TN tumor, whereas the prevalence in the literature is 80 and 14%, respectively. Binomial *p*-values for this are 0.486 and 0.203, respectively [51, 52]. Finally, the proportion of bilateral/contralateral breast cancer in the individuals of our cohort (*BRCA1* positive: 6 of 13, controls: 6 of 21) did also not differ significantly from the previously reported ones (44.1 and 17.2%, respectively) [53]. Binomial tests yielded a *p*-value of 1 and 0.157, respectively. These results indicate that the genetic and clinical data of our study population are without any obvious bias.

### Relative predictive performance of the data subgroups

**Fig. 2:**
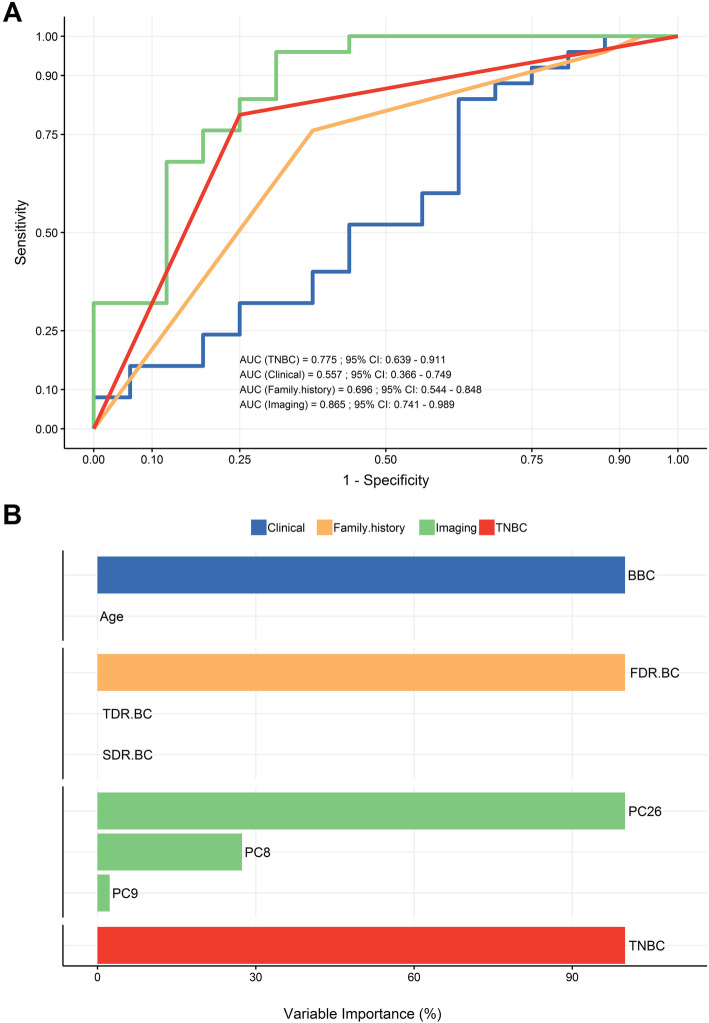
Predictive performance of TNBC, clinical, family history variables and imaging components **a** ROC analysis curve illustrating the relative predictive performance of clinical (blue) and family cancer history (orange) variables, TNBC (red) and imaging components (green) regarding genetic cancer risk estimation. Predictive power is measured by AUC. **b** Variable importance rankings for the two clinical variables (top), for the family cancer history variables and imaging components (middle) and for TNBC (bottom). Note that 3 imaging principal components, PC26, PC8 and PC9 are indicated as relevant to the prediction. For the abbreviations in the graphs see also “non-imaging features” in the section of Methods. CI, confidence interval

From the 41 available imaging principal components, the lasso regression selected 3 (PC26, PC8, PC9) as the most relevant for the classification (Fig. S4). These alone yielded an AUC performance of 0.86 (Fig. 2a and b). First, second and third-degree family cancer history was entered into a lasso regression within the same cross-validation regime as the imaging-based model yielding an overall AUC of 0.70 after selecting just one variable (FDR.BC) (Fig. 2a and b). TNBC alone yielded an AUC of 0.77 (Fig. 2a). Clinical components including Age and BBC showed a predictive value slightly better than chance (AUC 0.56) (Fig. 2a). BBC was ranked as the most important variable, relative to which, Age showed no predictive value (Fig. 2b) (see also supplementary Table S2). As an exploratory measure, we computed pairwise Spearman correlations between all variables used in the analysis. As expected, in this simpler analysis, we observed strong associative relationships of the target variable *BRCA* mutation status with TNBC and the imaging components. The correlation plot also uncovered less obvious but significant correlations emerged such as family history subcomponents with the imaging components (Fig. S6).

### Combining imaging with non-imaging features improves prediction performance

**Fig. 3:**
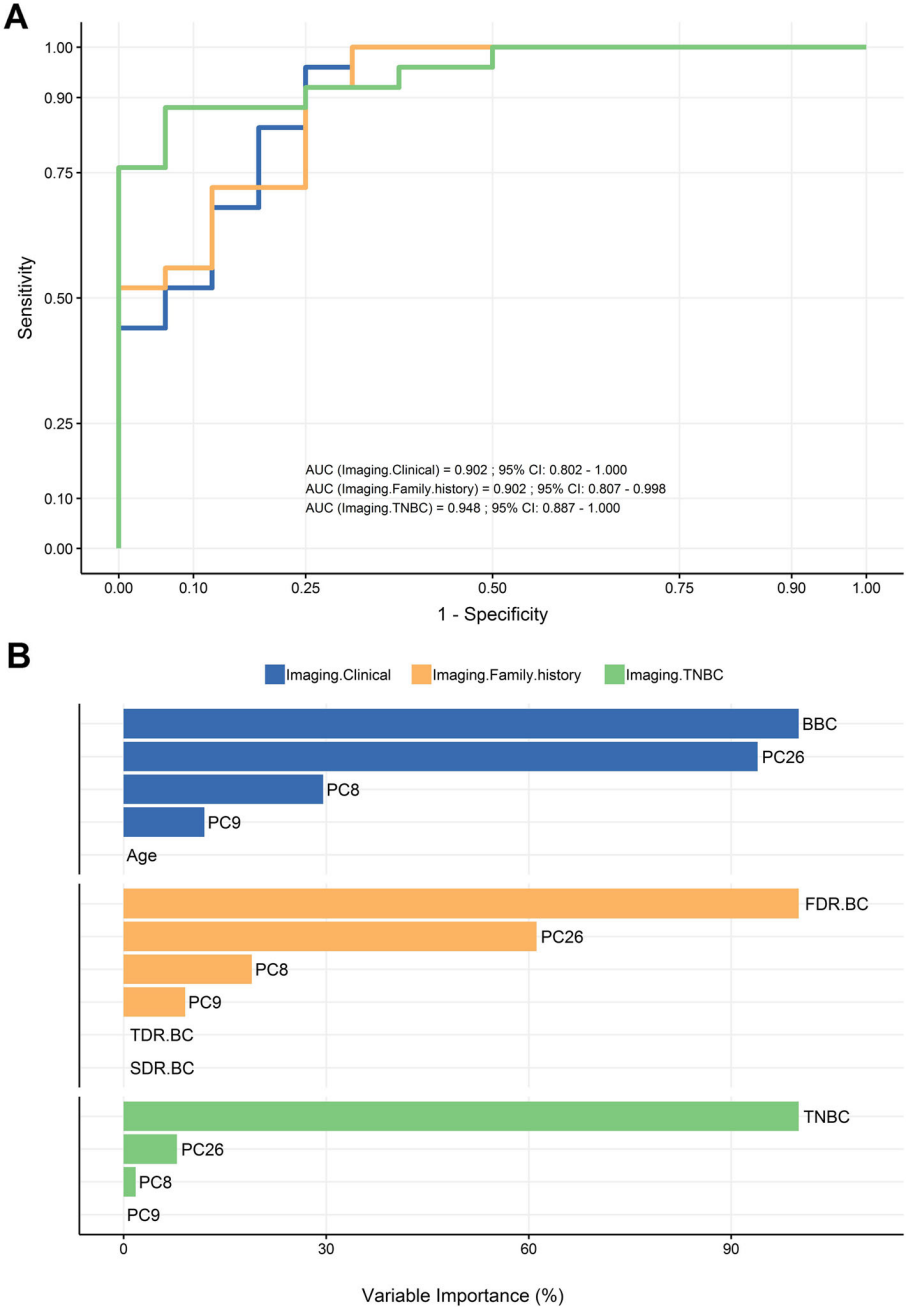
Improved predictive performance of non-imaging variables combined with imaging components **a** ROC analysis curve showing the relative AUC performance of the following variable combinations regarding genetic cancer risk: imaging components and clinical variables (blue), imaging components and family cancer history variables (orange), and finally imaging components and TNBC (green). **b** Variable importance rankings for the aforementioned variable subsets

We compared the classification performance after combination of imaging components with (i) TNBC, (ii) clinical variables and (iii) family cancer history variables (Fig. 3). From the three subsets the best predictive performance regarding *BRCA* status was observed when adding TNBC to the imaging components (AUC 0.94). TNBC was selected as the variable of highest importance, followed by just two of three imaging components (PC28, PC8) (Fig. 3a and b). Combining imaging with the clinical variables yielded an AUC of 0.90 with just BBC being retained along with the best 3 imaging components (Fig. 3a and b). Finally, the family history variables grouped together with imaging gave identical classification performance to imaging with clinical information (Fig. 3a). Out of all family cancer history variables lasso-regression model selected only the FDR.BC as the most important along with the 3 imaging components (Fig. 3b).

### Imaging can compensate the predictive value of family cancer history

Next, we investigated whether imaging can compensate the information about distant (2nd and 3rd degree) relatives (Fig. S7). To address this we contrasted AUC performance of the full set of variables, but excluding imaging information, against all variables, but excluding the cancer data of distant relatives from the family history. In the former scenario, the AUC was estimated at 0.82, whereas in the latter we obtained a much improved classification performance (AUC 0.95) (Fig. S7A). In both cases lasso model selected TNBC as the variable having the higher predictive power (Fig. S7B).

**Fig. 4:**
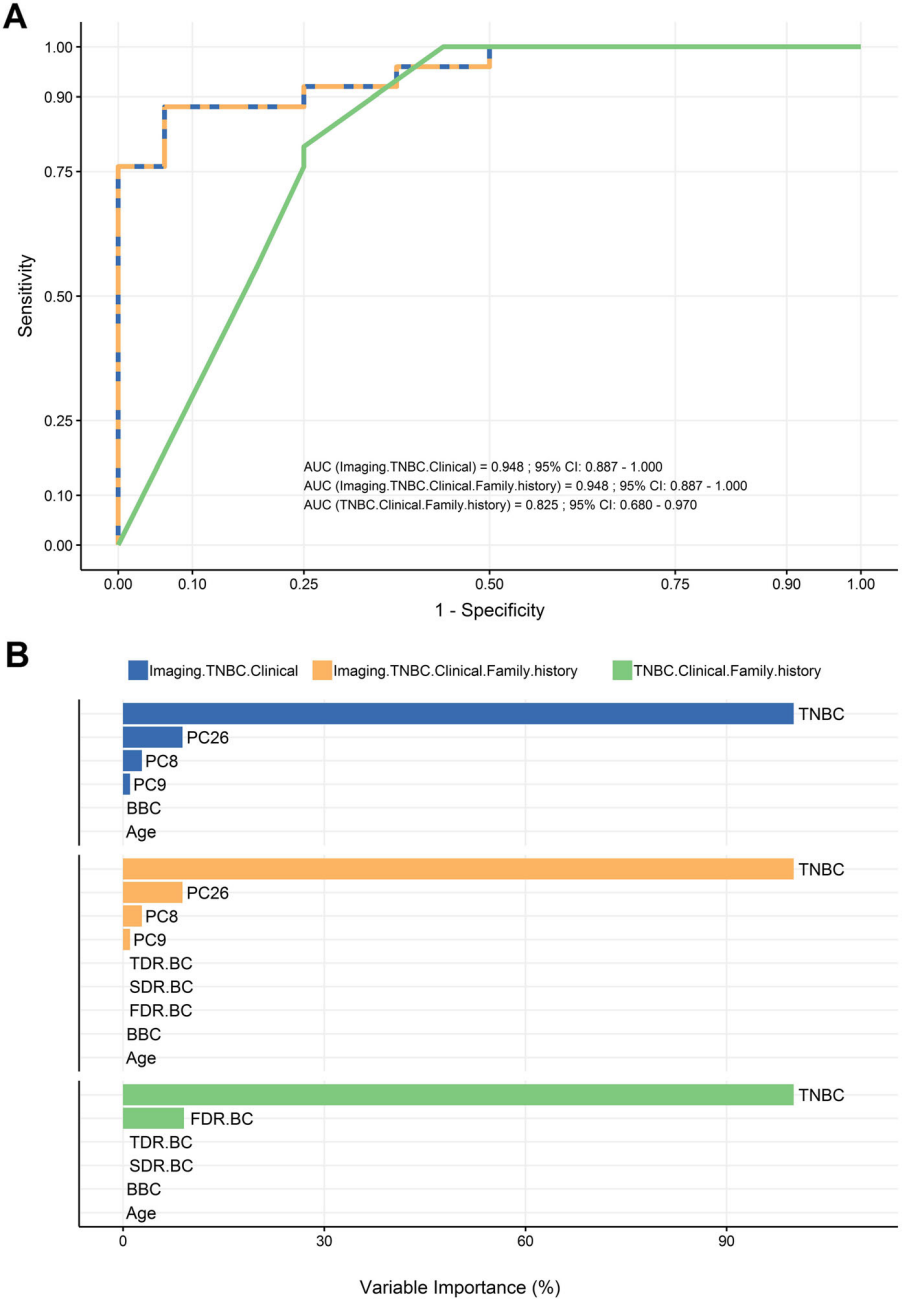
Imaging compensates the predictive power of family cancer profile **a** ROC analysis curve illustrating the relative AUC performance of the following combinations of variables: imaging components together with TNBC and clinical variables (blue), all available variables (orange) and TNBC together with clinical and family history variables (green). Note that the subset of variables without the imaging components showed the lowest predictive power and that the subset including imaging components has similar predictive value with the full set of variables. **b** Variable importance rankings for the aforementioned variable subsets, indicating TNBC and imaging components as the most important estimators of *BRCA* status

Further we assessed the algorithm performance when considering imaging as a proxy for the whole family cancer history (Fig. 4). The following combinations were analyzed i) all available genetic risk variables (TNBC, clinical, family history) except imaging, ii) all available variables (TNBC, clinical, imaging) except family history and iii) the full set of variables. The analysis, where imaging was excluded, resulted in the worst performance (AUC 0.82), with only TNBC and FDR.BC selected as relevant to the classification (Fig. 4a, b and Fig. S7A). When excluding all variables associated with family history, we gained an improvement in AUC to 0.95, with only TNBC and 3 imaging components showing considerable predictive power (Fig. 4a and b). Finally, when we supplied all available variables to the algorithm, we observed the same classification performance with the previous analysis (Fig. 4a). Similarly, importance ranking indicated that TNBC and imaging components captured the whole predictive power (Fig. 4b).

## Discussion

The significance of the identification of *BRCA1/2* pathogenic variants for the surveillance and design of personalized treatment with appropriate chemotherapy scheme, and prophylactic mastectomy/oophorectomy for the affected individuals is well established [54–56]. After the introduction of PARP (polyadenosine diphosphate ribose polymerase) inhibitors as a therapy of choice for *BRCA1/2*-related HBOC, the differentiation between genetic and sporadic breast cancer became increasingly important [57, 58]. Yet, the increased demand for genetic testing has been accompanied by a significant increase in health care costs, long waiting lists and delayed delivery of results [59, 60]. Psychosocial effects, as a result of pathological findings, demand additional support, further burdening the health system [61]. Nevertheless, many high genetic risk breast cancer cases still escape genetic screening, since prediction models, inclusion criteria and guidelines are mainly designed on the basis of subjective criteria such as the family cancer history [11, 62, 63]. Therefore, the accurate assessment of genetic risk and consequently the optimization of pre-selection for genetic testing by the clinicians are still ongoing and remain challenging.

To date, there are only few studies exploring the association of radiographic texture features with *BRCA* mutation status. However, all used computerized texture analysis on digital mammographs and focused on differentiating between healthy *BRCA1/2*-carriers and healthy non-carriers at high genetic risk or low-risk women [64–67]. To our knowledge the contribution of computer-extracted MRI texture features to the prediction *BRCA1/2*-associated genetic risk in already affected individuals has not been previously analyzed. Three imaging principal components out of the 41 studied here, namely PC28, PC8 and PC9, showed a relatively high predictive power. Interestingly, their combined performance surpassed that of family cancer history by more than 5% (Fig. 2a and b). This further supports their high value as *BRCA* estimators, since family cancer profile is considered the most eligible inclusion criterion for genetic testing as well as a main parameter used by many commercially available *BRCA*-prediction models [11, 68, 69]. FDR.BC was highlighted as the top family history variable in importance ranking (Fig. 2b). Nevertheless, the predictive performance of  ovarian cancer (OV) parameters could not be evaluated due to the small proportion of OV affected relatives of the studied cases (supplementary Table S1). TNBC alone was strongly correlated with the prediction of *BRCA* deleterious variants, confirming previous studies establishing TNBC as a powerful independent selection criterion for screening (Fig. 2a, supplementary Table S2) [6, 7].

Somewhat unexpectedly, age at disease onset and BBC, while commonly assigned as genetic risk estimators [2], showed a relatively low predictive strength, with BBC capturing the whole predictive power in our cohort (Fig. 2a and b). BBC has been previously linked to the prediction of *BRCA1* [69, 70], potentially suggesting that the unequal proportion of *BRCA1* compared to *BRCA2* affected individuals (13 to 3) in the current study, which however is in accordance with the distribution in literature, could explain this finding. Larger studies analyzing pure *BRCA1* and *BRCA2* cohorts are necessary to provide further insight into the role of BBC as predictor. The unusually low importance of Age in the prediction model in contrast to previous reports [9, 69, 70], may at least in part be attributed to the design of our relative small pilot study that included only breast cancer cases at high genetic risk. The mean age of disease onset was similar in both groups. Based though on the established inclusion criteria only 10% of female individuals receiving genetic testing due to the early onset breast cancer alone (BC at age < 36 years with negative family history) are expected to carry a *BRCA1/2* pathogenic variant [2]. This estimation may lend empirical strengthen to the hypothesis that Age alone is indeed a weak risk estimator. It is also likely that our regression model, which contains and estimates at once a much wider range of variables, compensates for correlations between Age and other variables that would not be accounted for in previous, one-variable-at-a-time studies.

Next, we investigated whether imaging components along with the non-imaging variables, a priori known to be relevant for HBOC, could complementarily improve genetic risk estimation (Fig. 3). The Lasso-principal component regression model yielded the strongest classification performance when imaging and TNBC were combined, with TNBC driving the prediction (Fig. 3a and b). The determination of tumor receptor status is nowadays part of the medical routine due to its importance for therapy decision-making and prognosis [71, 72]. On the other hand, breast MRI has been lately progressively incorporated into diagnosis [15, 73]. Since both histopathological examination and MR imaging results are objective parameters in contrast to the more subjective family cancer history, their combined evaluation along with texture analysis, could establish a new promising estimator for optimizing genetic risk assessment. The added value of imaging components was also highlighted by the substantial improvement of the predictive performance for both family cancer history and clinical variables (Fig. 3a).

HBOC due to germline *BRCA1/2* deleterious variants follows an autosomal dominant inheritance, so that the identification of many affected relatives in a three-generation pedigree is expected. For this reason, the positive family history represents the gold-standard estimator for genetic evaluation [2, 9, 11]. Our analysis also confirmed the strong correlation with *BRCA* status (Fig. 2a). Nevertheless, a significant number of breast cancer cases at high genetic risk do not receive genetic screening as a result of missing family history data or non-informative genealogical family trees. Adoption, smaller families, few female and excess of male relatives as well as the premature mortality of female relatives together with the incomplete penetrance of the disease are common reasons for failure to identify individuals at risk [12]. Taking these limitations into consideration, we examined whether the variables relating to family cancer history could be replaced by imaging components in the prediction model. When we performed the analysis by excluding either the cancer data of distant relatives or imaging we observed that the latter could supplant the information on distant family cancer history and improve the prediction power (Fig. S7A). TNBC and imaging components drove the prediction to the extent that the lasso model selection zeros clinical and 1rd degree family history variables out in choosing the best model for classification performance (Fig. S7B). By replacing all family history variables with imaging components, we gained an approximately 4% improvement of the predictive performance, which was similar to the predictive power of all available parameters tested (Fig. 4a). Again, TNBC and imaging components dominated clinical und family variables (Fig. 4b), indicating that imaging information suffices, irrespective of whether family history is included or not in the model. Collectively, these results suggest that imaging data compensates the information arising from family profile, emphasizing it’s value as independent predictive factor.

The most important limitation of this study is the relative low number of subjects. Indeed the 95% AUC confidence intervals reported suggest that conducting formal hypothesis tests between the relative performance of the different data modes are unlikely to prove significantly different. However, we should not overlook the fact that these preliminary results are based on state-of-the-art statistical lasso techniques that are designed to compensate for the uncertainty necessitated by small subject groups when large numbers of variables are available for each. Furthermore, the family of lasso-based techniques come with robust mathematical guarantees that the selected parsimonious model, and the associated learned beta coefficients represent the true underlying model even in the scenario when number of subjects (N) < < total number of potential variables (p). These methods also offer the unique opportunity to consider the impact of fitting a much larger set of features all at once in addressing these kinds of clinical prediction problems. In comparison standard regression-based techniques would be overwhelmed by the scenario of when N < < p.

## Conclusions

In this study we presented preliminary results showing that imaging texture features extracted from T1 weighted breast MRIs can serve as a predictor allowing for differentiation between high risk breast cancer individuals with or without *BRCA1/2* variants. Incorporation of imaging components to the prediction model, along with other established *BRCA*-related genetic risk estimators, considerably adds to the likelihood of ascertainment of HBOC carriers, potentially enabling a more efficient decision-making for genetic screening. The combination of imaging components and triple negative receptor status was indicated as the most important estimator. On the other hand, we provided a new glimpse about prioritization of clinical findings by showing clues that clinical genetic risk predictors such as the age of disease onset could have indeed a weak possibly lower value in estimating genetic risk as was originally though. Finally, in the common scenario of missing family information, imaging components can compensate the lack of family data, thus improving assessment by the clinicians and geneticists. Our study, similar to previous ones, highlights that the hidden information of MRI modalities is noteworthy and should not be overlooked. In this case, integration of computer-extracted MRI textures in the radiologist routine diagnosis could prove a cost-effective way for optimization of prediction models and selection criteria for *BRCA1/2* testing, since it does not add significantly to the cost of radiologic imaging. Finally, lasso based methods allow the compensation of the small size sample by the large numbers of variables/features tested. This can facilitate the publication of reliable results not only from large cohorts but also from smaller studies and enable fast systematic reviews. Nevertheless, we acknowledge that further studies in larger cohorts and comparisons not only between high risk genetic breast cancer carriers and not carriers but also with low-risk affected women is warranted in order to confirm and strengthen the role of MRI texture features as predictor for *BRCA* status.

## Supplementary information

**Additional file 1: Supplementary file.** PDF file containing **Supplementary methods.** Detailed description of Lasso-principal component regression. **Fig. S1.** Description of the work-flow. **Fig. S2.** Description of the MR image annotation steps. **Fig. S3.** (A) Visual representation of the annotation sampling process. (B) Construction of patches around each seed point. **Fig. S4.** Plot showing the amount of variance explained by each of the imaging principal components supplied to the Lasso regression algorithm. **Fig. S5.** Comparison of L-PCR with two other widely used penalisation approaches, namely ridge regression and elastic net, as applied to the full dataset. **Fig. S6.** Exploratory Correlation Plot showing the relative correlations strengths between the different variables used in the study and between those variables and the *BRCA* mutation status (MS). **Fig. S7.** (A) ROC analysis curve comparing the relative predictive performance of two subsets of data, full set of variables excluding only cancer information for distant 2nd and 3rd relatives (blue) against all variables excluding imaging (orange). (B) Variable importance for the two aforementioned subsets. **Table S1.** Mutation status, clinical, histopathological and family structure information for 16 *BRCA1/2* individuals and 25 controls used in the study. **Table S2.** Classification results of Fig. 2. Confusion matrices and associated diagnostics for each group of variables individually and in combinations. CI, confidence interval

## Data Availability

The data supporting this article are provided in the supplementary files available from the publisher’s website. Also they are available from the authors. The implementation of the algorithms used in the manuscript is available at the following URL: https://github.com/spakcjl/BRCA_LassoCode

## Abbreviations

TNBC: Triple negative receptor status
L-PCR: Lasso-principal component regression
AUC: Area under the curve
MRI: Magnetic resonance imaging
HBOC: Hereditary breast and ovarian cancer
EP: Empirical probability
ROI: Region of interest
FDR.BC: 1st degree relatives with breast cancer
FDR.OC: 1st degree relatives with ovarian cancer
SDR.BC: 2nd degree relatives with breast cancer
SDR.OC: 2st degree relatives with ovarian cancer
TDR.BC: 3rd degree relatives with breast cancer
TDR.OC: 3rd degree relatives with ovarian cancer
MBC: Male breast cancer
PCA: Principal component analysis
ROC: Receiver operating characteristic
PARP: Polyadenosine diphosphate ribose polymerase
