# Supplementary material for: Breast MRI texture analysis for prediction of BRCA-associated genetic risk
Source: BMC Med Imaging. 2020 Jul 29;20:86. doi: 10.1186/s12880-020-00483-2 (PMC7388478; doi:10.1186/s12880-020-00483-2)
Supplement: Supplementary file 1 — Additional file 1: Supplementary file. PDF file containing Supplementary methods. Detailed description of Lasso-principal component regression. Fig. S1. Description of the work-flow. Fig. S2. Description of the MR image annotation steps. Fig. S3. (A) Visual representation of the annotation sampling process. (B) Construction of patches around each seed point. Fig. S4. Plot showing the amount of variance explained by each of the imaging principal components supplied to the Lasso regression algorithm. Fig. S5. Comparison of L-PCR with two other widely used penalisation approaches, namely ridge regression and elastic net, as applied to the full dataset. Fig. S6. Exploratory Correlation Plot showing the relative correlations strengths between the different variables used in the study and between those variables and the BRCA mutation status (MS). Fig. S7. (A) ROC analysis curve comparing the relative predictive performance of two subsets of data, full set of variables excluding only cancer information for distant 2nd and 3rd relatives (blue) against all variables excluding imaging (orange). (B) Variable importance for the two aforementioned subsets. Table S1. Mutation status, clinical, histopathological and family structure information for 16 BRCA1/2 individuals and 25 controls used in the study. Table S2. Classification results of Fig. 2. Confusion matrices and associated diagnostics for each group of variables individually and in combinations. CI, confidence interval [file 12880_2020_483_MOESM1_ESM.docx]

**Breast MRI texture analysis for prediction of BRCA-associated genetic risk**

Georgia Vasileiou^1*^ ^#^, Maria J. Costa^2*^, Christopher Long^2*^, Iris R. Wetzler^3^, Juliane Hoyer^1^, Cornelia Kraus^1^, Bernt Popp^1^, Julius Emons^4^, Marius Wunderle^4^, Evelyn Wenkel^3^, Michael Uder^3^, Matthias W. Beckmann^4^, Sebastian M. Jud^4^, Peter A. Fasching^4^, Alexander Cavallaro^3^, André Reis^1^, Matthias Hammon^3^

**SUPPLEMENTARY METHODS**

**Detailed description of Lasso-principal component regression**

Specifying a statistical framework appropriate to this problem requires combining imaging data comprising many voxels with subject and clinical attributes a priori known to be relevant to either the disease or the genetic status of the individual. Since imaging data by its nature is spatially highly correlated, we have developed an estimation regime that can mitigate the attendant collinearity or redundancy between the imaging predictors while selecting those variables, or groups of variables that can maximize predictive power. We developed a two-stage Principal Components L^1^-Penalized Logistic Regression due to the vastly larger number of predictors at hand compared to the number of subjects available. This performs in the first step an initial dimensionality reduction by constructing the first *M* principal components from the imaging data, **X**. =(X_1_…X_M_) and using these as predictors in the following linear regression model that is penalized using an L^1^-penalty:


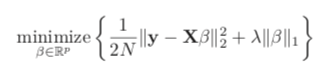


During this fit, the algorithm selects a small subset of those most relevant components as predictors in a regression model and combines them with clinical and subject relevant information. While the principal components transformation results in a dramatic reduction of features, parameter identifiability and overfitting issues will still persist unless additional model penalization in some form is performed. Lasso or L^1^-penalised regression offers the ability to simultaneously consider all variables and choose an optimal sparse subset of model predictors (components) based on their discriminant power while at the same time learning the Beta parameters. Compared to other competing variable selection alternatives, such as forward and backward selection/elimination methods lasso penalization can mitigate bias caused by variable ordering and the need to correct for p-values consequent to the multiple comparisons problem associated with sifting through potentially thousands of candidate models. Additionally, computing lasso models using principal components, where the predictors are orthogonal, improves the overall efficiency of the regression. Taken together, these factors serve to motivate L^1^-penalized principal component logistic regression as a well-specified method for this particular application.

For clarity and to give a wider context about these types of penalized regression techniques, we might add that the lasso penalty can be extended into a kind of mixed-norm framework known as the elastic net penalty that comprises of both an L^1^-and L^2^-penalized cost function that also compensates for situations where there are a large number of variables relative to the number of subjects. This is achieved by combining the lasso (L^1) penalty that performs an automatic model selection, while the L^2^ component tends to choose groups of parameters that are most correlated, and then shrinks those similar variables towards one another (the grouping property) [47]. In particular, we solve the following convex likelihood function specified as follows:


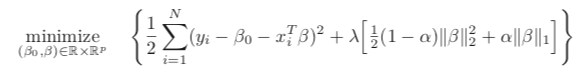


Two hyper-parameters need to be chosen in elastic net regression problems. Lambda controls the extent to which the model is penalised; a Lambda of 0 reduces the estimate to an unpenalized and perhaps unidentifiable ordinary least squares estimate whereas a large Lambda will result in a heavily penalised model with small coefficient vectors. The second hyper-parameter is alpha, which governs the amount of interplay within the penalty, between the model selection and the assumed correlation between the parameters. A choice of alpha close to 1 will tend to focus on a highly sparse model selection and will tend to ignore potential groupings between the parameters. Conversely, a choice of alpha close to zero will lead to the inclusion of all parameters and will tend to exploit correlations across a larger number of parameters.

**SUPPLEMENTARY FIGURES**


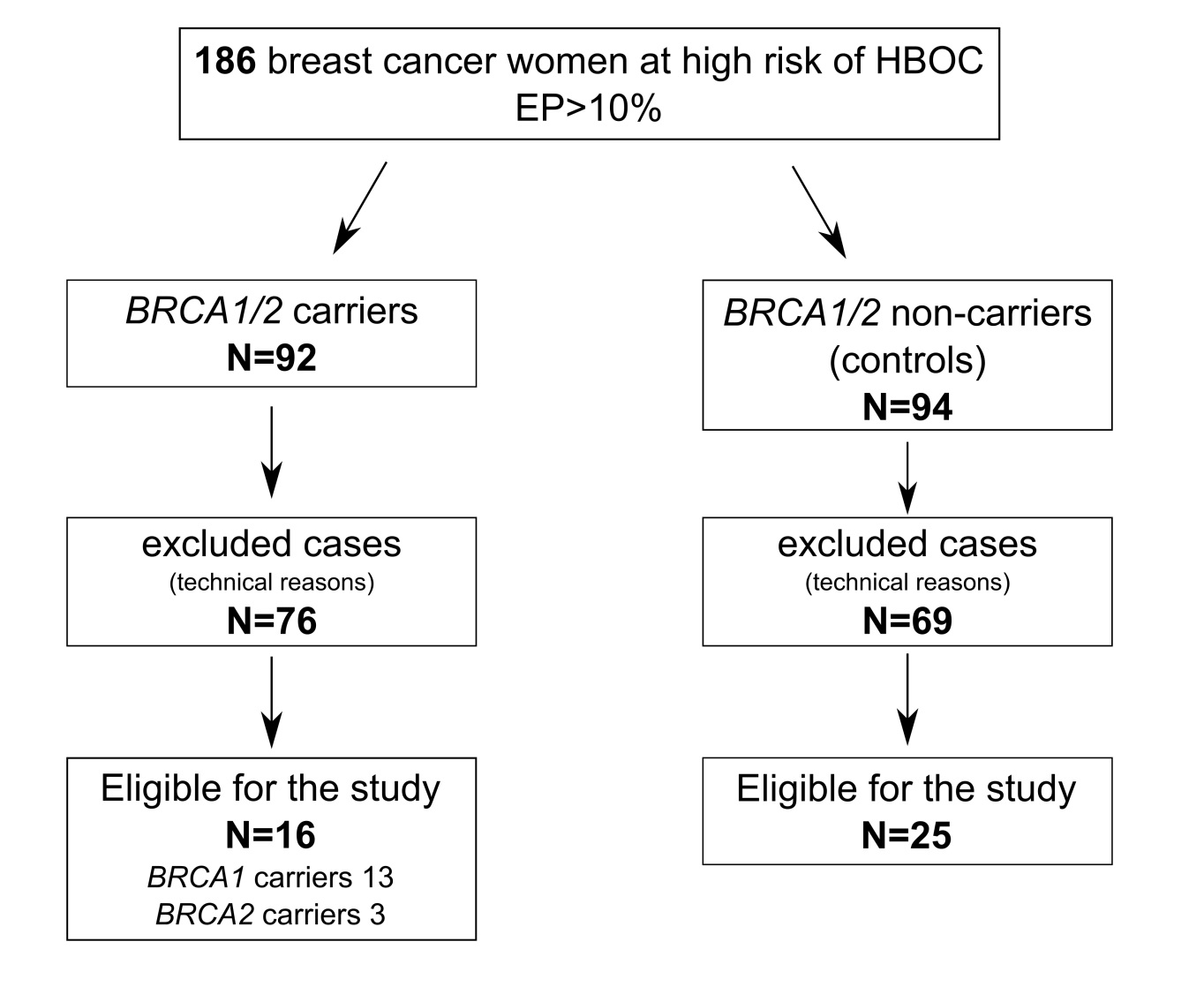


**Fig. S1.** Description of the work-flow.

| 1. After starting the annotation tool, the selected 3D image volume is opened | 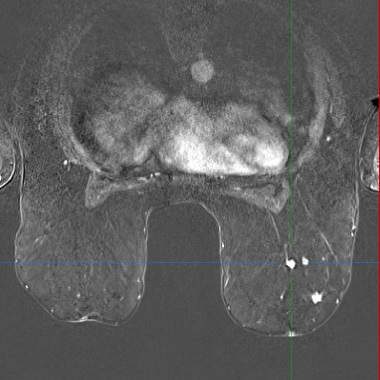 |
| --- | --- |
| 1. A new 3D segmentation mask is created for each malignant tumor in the image. | 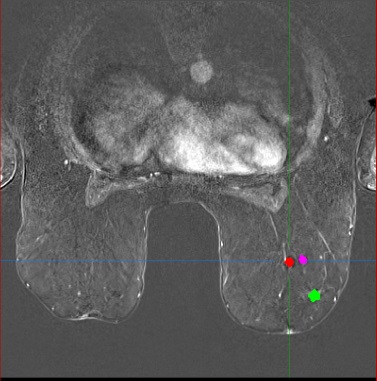 |
| 1. Several selection tools can be used to facilitate the creation of the region of interest around the tumor. | 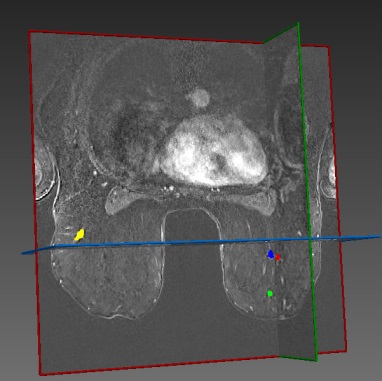 |
| 1. Each newly created mask can be saved as a binary image. |  |

**Fig. S2**. Description of the MR image annotation steps.


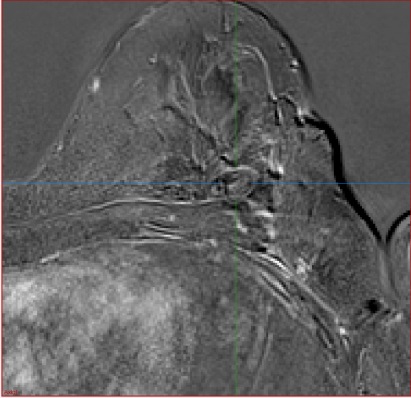

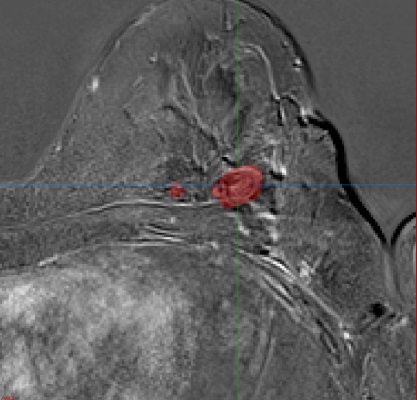

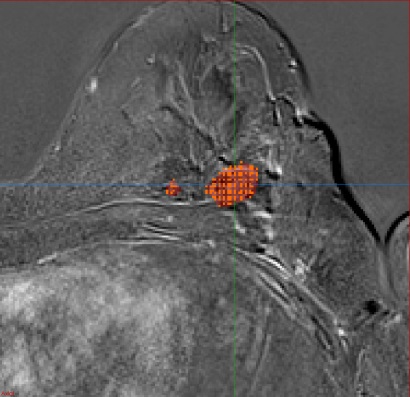


**A**

**B**


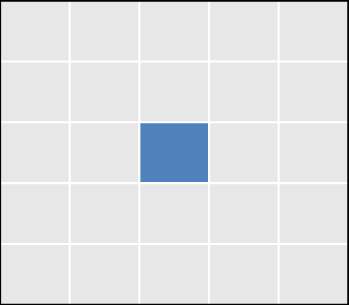


**65 image voxels**

**65 image voxels**

**seed**

**point**

**Fig. S3**. (A)The lesions in the original MR image (left) are annotated in the form of a mask (center, red overlay). This mask is then sampled at uniformly distributed locations (right). (B) Using seed points for intensity patch extraction from lesions in MR images, patch size 65x65 voxels.





**Fig. S4**. Plot showing the amount of variance explained by each of the imaging principal components supplied to the Lasso regression algorithm. As expected, there is an ordered decrease in the importance of each component as the number of components increases.


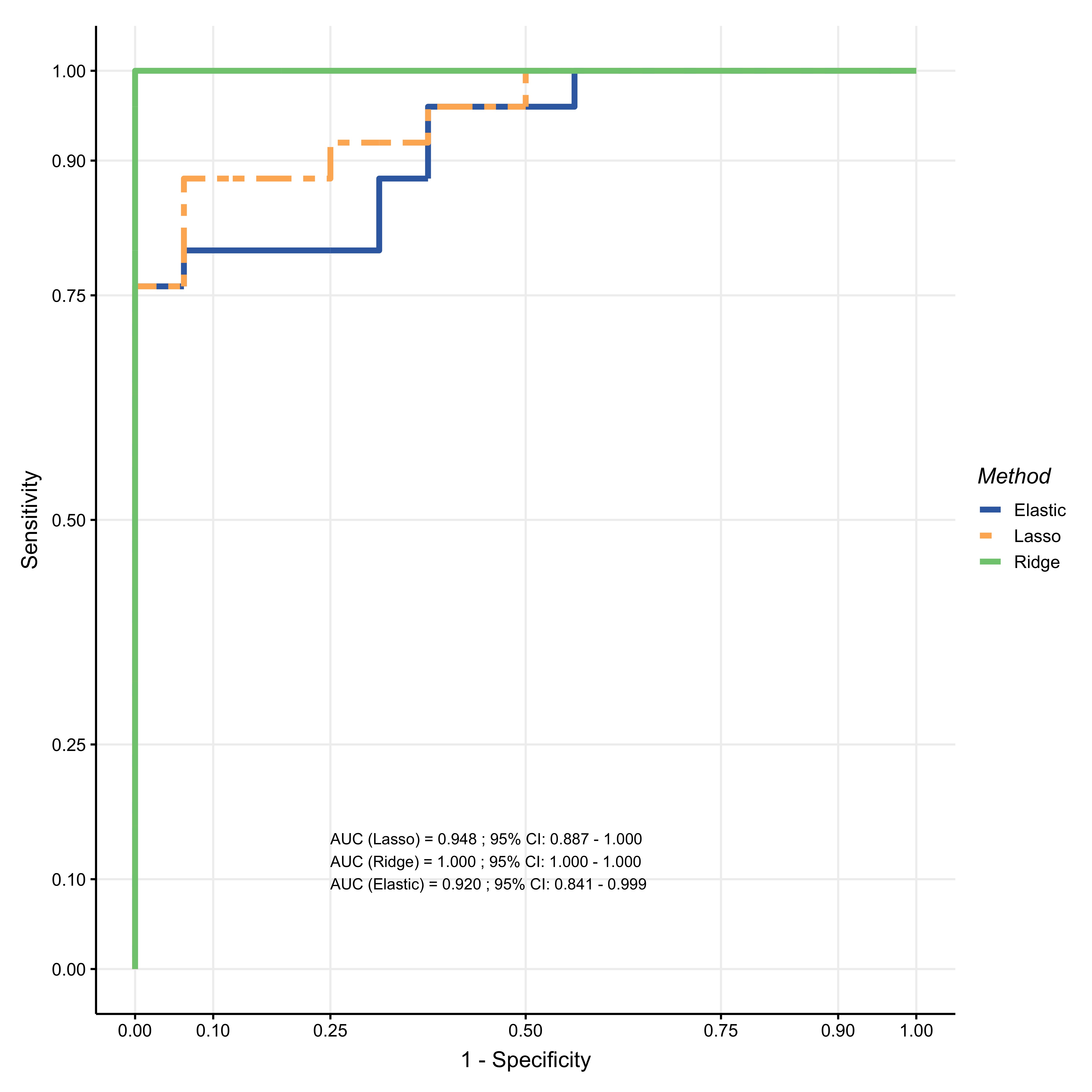


**Fig. S5**. Plot comparing three algorithms as applied to the full dataset, containing all modes of data (imaging+clinical+family history). Essentially, we contrast our L-PCR approach against two widely used and related penalisation approaches namely the L2 or ridge regression and a mixed norm or weighted L1+L2 approach, the elastic net. We see hat the benefits of variable selection intrinisic to our L-PCR approach outweigh somewhat the inclusion of an L2 norm which includes all variables in the final model leading to an overfitting problem that appears in the form of an overly optimistic ROC curve. The elastic net does better, but the grouping property of the elastic net turns out to be less beneficial than performing PCA followed by L1 penalisation as in L-PCR.


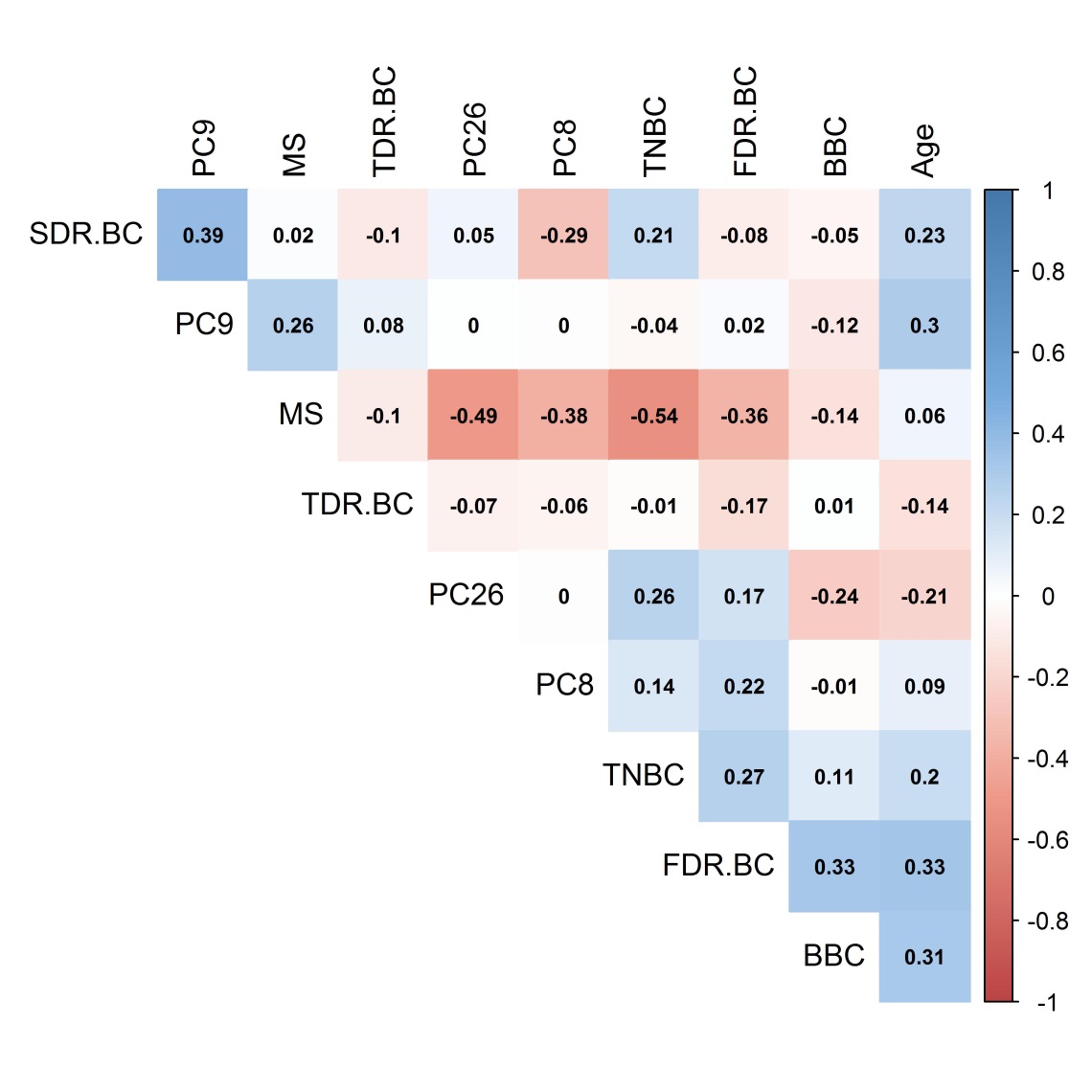


**Fig. S6.** Exploratory Correlation Plot showing the relative correlations strengths between the different variables used in the study and between those variables and the *BRCA* mutation status (MS). Blue and red coloring indicate positive and negative correlations respectively.


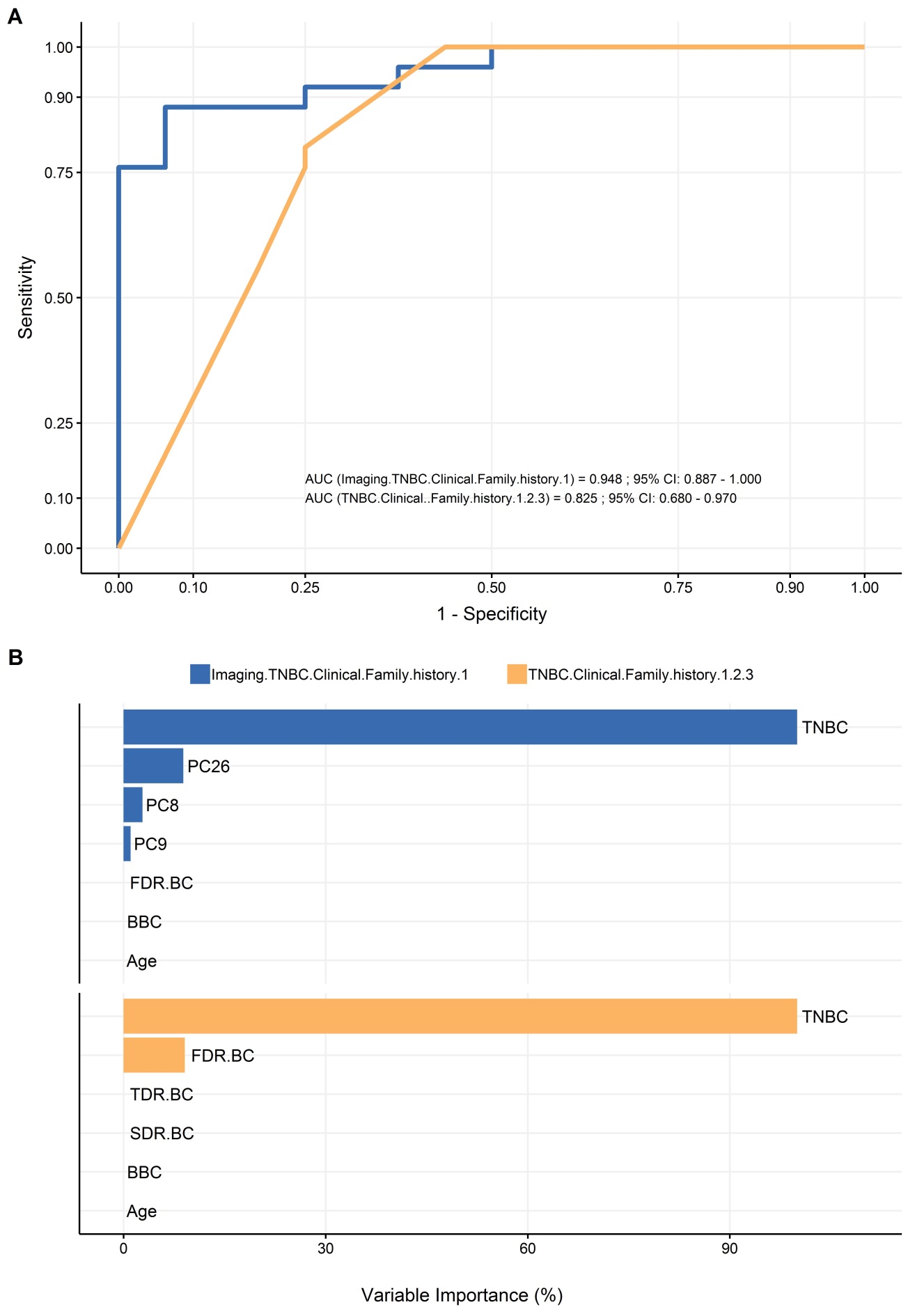
**Fig. S7.** (A) ROC analysis curve comparing the relative predictive performance of two subsets of data, full set of variables excluding only cancer information for distant 2^nd^ and 3^rd^ relatives (blue) against all variables excluding imaging (orange). (B) Variable importance for the two aforementioned subsets.

| **Nr.** | **Individual ID** | **Mutation**  **status** | **Age** | **FDR.BC** | **FDR.OC** | **SDR.BC** | **SDR.OC** | **TDR.BC** | **TDR.OC** | **MBC** | **EP** | **BBC** | **TNBC** |
| --- | --- | --- | --- | --- | --- | --- | --- | --- | --- | --- | --- | --- | --- |
| **1** | 230002AWEI | BRCA1 | 52 | 4 | 0 | 0 | 0 | 0 | 0 | 0 | 30.7 | 0 | 1 |
| **2** | 230005P8DP | BRCA1 | 29 | 1 | 0 | 1 | 0 | 0 | 0 | 0 | 30.7 | 0 | 1 |
| **3** | 23000EAP6I | BRCA1 | 43 | 2 | 0 | 0 | 0 | 0 | 0 | 0 | 30.7 | 1 | 1 |
| **4** | 23000LHVT5 | BRCA1 | 47 | 1 | 0 | 1 | 0 | 0 | 0 | 0 | 30.7 | 1 | 1 |
| **5** | 23000QQ3MI | BRCA1 | 32 | 1 | 0 | 0 | 0 | 0 | 0 | 0 | 30.7 | 1 | 1 |
| **6** | 23000SS2OS | BRCA1 | 35 | 1 | 0 | 0 | 0 | 0 | 0 | 0 | 19.3 | 0 | 1 |
| **7** | 23000W4JGK | BRCA1 | 44 | 1 | 0 | 0 | 0 | 0 | 0 | 0 | 30.7 | 1 | 1 |
| **8** | 23000WW04O | BRCA1 | 41 | 1 | 0 | 0 | 0 | 0 | 0 | 0 | 19.3 | 0 | 1 |
| **9** | 23000YY8QM | BRCA1 | 40 | 0 | 1 | 1 | 1 | 0 | 2 | 0 | 48,4 | 0 | 1 |
| **10** | 23000ZZG3Z | BRCA1 | 30 | 0 | 0 | 0 | 0 | 1 | 0 | 0 | 30.7 | 1 | 1 |
| **11** | 23000ZZNNJ | BRCA1 | 33 | 0 | 0 | 0 | 0 | 0 | 0 | 0 | 11.2 | 0 | 1 |
| **12** | 23000XXLD9 | BRCA1 | 39 | 1 | 0 | 0 | 0 | 2 | 0 | 0 | 30.7 | 1 | 1 |
| **13** | 230008G9KO | BRCA1 | 34 | 0 | 0 | 0 | 0 | 0 | 0 | 0 | 10.1 | 0 | 0 |
| **14** | 2300011C95 | BRCA2 | 28 | 1 | 0 | 0 | 0 | 0 | 0 | 0 | 10.1 | 0 | 0 |
| **15** | 2300011NP1 | BRCA2 | 32 | 0 | 0 | 0 | 0 | 2 | 0 | 0 | 30.7 | 0 | 0 |
| **16** | 230004C0SW | BRCA2 | 30 | 0 | 0 | 0 | 0 | 0 | 0 | 0 | 10.1 | 0 | 0 |
| **17** | 230000K10K | control | 34 | 0 | 0 | 0 | 0 | 0 | 0 | 0 | 10.1 | 0 | 0 |
| **18** | 230001LM9L | control | 50 | 0 | 0 | 0 | 0 | 0 | 0 | 0 | 24.8 | 1 | 0 |
| **19** | 230002YRI2 | control | 32 | 0 | 0 | 0 | 0 | 0 | 0 | 0 | 10.1 | 0 | 0 |
| **20** | 230004014C | control | 28 | 0 | 0 | 0 | 0 | 0 | 0 | 0 | 11.2 | 0 | 1 |
| **21** | 2300044IKO | control | 44 | 1 | 0 | 0 | 0 | 0 | 0 | 0 | 30.7 | 1 | 0 |
| **22** | 230007FNNR | control | 19 | 0 | 0 | 0 | 0 | 0 | 0 | 0 | 10.1 | 0 | 0 |
| **23** | 230007R7ZJ | control | 36 | 0 | 0 | 1 | 0 | 1 | 0 | 0 | 30.7 | 0 | 0 |
| **24** | 23000BBGBF | control | 54 | 0 | 0 | 1 | 0 | 0 | 0 | 0 | 24.8 | 1 | 0 |
| **25** | 23000BJ67B | control | 25 | 0 | 0 | 0 | 0 | 0 | 0 | 0 | 10.1 | 0 | 0 |
| **26** | 23000DDTTP | control | 27 | 0 | 0 | 0 | 0 | 0 | 0 | 0 | 10.1 | 0 | 0 |
| **27** | 23000I246Q | control | 40 | 0 | 0 | 0 | 0 | 1 | 0 | 0 | 11.2 | 0 | 1 |
| **28** | 23000I286I | control | 29 | 0 | 0 | 0 | 0 | 0 | 0 | 0 | 10.1 | 0 | 0 |
| **29** | 23000IQ8Y2 | control | 33 | 0 | 0 | 0 | 0 | 1 | 0 | 0 | 19.3 | 0 | 0 |
| **30** | 23000NJWNR | control | 43 | 1 | 0 | 0 | 0 | 0 | 0 | 0 | 30.7 | 1 | 0 |
| **31** | 23000P9B5L | control | 30 | 0 | 0 | 0 | 0 | 0 | 1 | 0 | 48.4 | 0 | 0 |
| **32** | 23000QMM6I | control | 49 | 0 | 0 | 1 | 0 | 0 | 0 | 0 | 11.2 | 0 | 1 |
| **33** | 23000RB57R | control | 35 | 2 | 0 | 0 | 0 | 0 | 1 | 0 | 30.7 | 1 | 0 |
| **34** | 23000TD61L | control | 46 | 1 | 0 | 0 | 0 | 0 | 0 | 0 | 30.7 | 1 | 0 |
| **35** | 23000VFJJV | control | 33 | 0 | 0 | 0 | 0 | 0 | 0 | 0 | 10.1 | 0 | 0 |
| **36** | 23000VVO3Z | control | 47 | 1 | 0 | 0 | 0 | 0 | 0 | 0 | 9.2 | 0 | 0 |
| **37** | 23000YYV6Q | control | 32 | 0 | 0 | 0 | 0 | 2 | 0 | 0 | 30.7 | 0 | 0 |
| **38** | 230001XV9H | control | 54 | 1 | 0 | 1 | 0 | 0 | 0 | 0 | 30.7 | 0 | 0 |
| **39** | 230006Q76Q | control | 68 | 0 | 0 | 0 | 0 | 0 | 0 | 0 | 11.2 | 0 | 1 |
| **40** | 23000HPR51 | control | 35 | 0 | 0 | 0 | 0 | 0 | 0 | 0 | 10.1 | 0 | 0 |
| **41** | 23000L56TD | control | 27 | 0 | 0 | 1 | 0 | 0 | 0 | 0 | 11.2 | 0 | 1 |

**Table S1.** Mutation status, clinical, histopathological and family structure information for 16 *BRCA1/2* individuals and 25 controls used in the study.

| **TNBC** | | Reference | |
| --- | --- | --- | --- |
|  |  | Control | *BRCA1/2* |
| Prediction | Control | 20 | 4 |
|  | *BRCA1/2* | 5 | 12 |
| Accuracy (95% CI): 0.780 (0.624, 0.894)  Sensitivity: 0.75, Specificity: 0.80  Positive Predictive Value: 0.706, Negative Predictive Value: 0.833  Balanced Accuracy: 0.775 | | | |
| **Clinical variables** | | Reference | |
|  |  | Control | *BRCA1/2* |
| Prediction | Control | 15 | 9 |
|  | *BRCA1/2* | 10 | 7 |
| Accuracy (95% CI): 0.536 (0.374, 0.693)  Sensitivity: 0.438, Specificity: 0.600  Positive Predictive Value: 0.412, Negative Predictive Value: 0.625  Balanced Accuracy: 0.519 | | | |
| **Family history variables** | | Reference | |
|  |  | Control | *BRCA1/2* |
| Prediction | Control | 19 | 12 |
|  | *BRCA1/2* | 6 | 4 |
| Accuracy (95% CI): 0.756 (0.597, 0.876)  Sensitivity: 0.750, Specificity: 0.760  Positive Predictive Value: 0.667, Negative Predictive Value: 0.826  Balanced Accuracy: 0.755 | | | |
| **MRI-derived imaging measures** | | Reference | |
|  |  | Control | *BRCA1/2* |
| Prediction | Control | 21 | 4 |
|  | *BRCA1/2* | 4 | 12 |
| Accuracy (95% CI): 0.805 (0.651, 0.912)  Sensitivity: 0.750, Specificity: 0.840  Positive Predictive Value: 0.750, Negative Predictive Value: 0.840  Balanced Accuracy: 0.795 | | | |
| **Combined data modalities** | | Reference | |
|  |  | Control | *BRCA1/2* |
| Prediction | Control | 21 | 15 |
|  | *BRCA1/2* | 4 | 1 |
| Accuracy (95% CI): 0.878 (0.738, 0.959)  Sensitivity: 0.938, Specificity: 0.840  Positive Predictive Value: 0.790, Negative Predictive Value: 0.955  Balanced Accuracy: 0.889 | | | |

**Table S2.** Classification results of Figure 2. Confusion matrices and associated diagnostics for each group of variables individually and in combinations. CI, confidence interval.
